# A retrospective review of rabies post-exposure prophylaxis queries, South Africa, 2016–2019

**DOI:** 10.4102/sajid.v37i1.354

**Published:** 2022-09-13

**Authors:** Trisha A. Whitbread, Kathleen J. Kabuya, Nimesh Naran, Amilcar M. Juggernath, Moushumi A. Mathews, Lucille H. Blumberg, Jacqueline Weyer, Vivien Essel

**Affiliations:** 1 Outbreak Response Unit, Division of Public Health Surveillance, National Institute for Communicable Diseases, Johannesburg, South Africa; 2 Department of Community Health, School of Public Health, Faculty of Health Sciences, University of the Witwatersrand, Johannesburg, South Africa; 3 Department of Public Health Medicine, Charlotte Maxeke Johannesburg Academic Hospital, Gauteng Department of Health, Johannesburg, South Africa; 4 Centre for Emerging Zoonotic and Parasitic Diseases, and Division of Public Health Surveillance, National Institute for Communicable Diseases, Johannesburg, South Africa; 5 Faculty of Veterinary Science, University of Pretoria, Pretoria, South Africa; 6 Department of Medical Virology, Faculty of Health Sciences, University of Pretoria, Pretoria, South Africa; 7 Department of Microbiology and Infectious Diseases, Faculty of Health Sciences, University of the Witwatersrand, Johannesburg, South Africa

**Keywords:** rabies, post-exposure prophylaxis, NICD, clinician hotline, animal exposure

## Abstract

**Background:**

The National Institute for Communicable Diseases (NICDs) of South Africa (SA) provides technical support to healthcare workers (HCWs) with regard to infectious diseases through the NICD clinician hotline. Queries to the hotline are often about rabies prophylaxis. An analysis of these queries may help to identify knowledge gaps amongst HCWs regarding prevention of rabies in humans in SA.

**Methods:**

A retrospective descriptive review was conducted to analyse rabies post-exposure prophylaxis (PEP) queries received by the NICD from 01 January 2016 to 31 December 2019.

**Results:**

A total of 4655 queries were received by the NICD clinician hotline for the study period, of which 2461 pertained to rabies PEP (52.87%). The largest number of calls were placed by HCWs (*n* = 2313/2437; 94.9%). Queries originated mainly from Gauteng (*n* = 912/2443; 37.3%) and KwaZulu-Natal (*n* = 875/2443; 35.8%) provinces. A total of 50 different types of animals were related to exposures involving humans. Dogs (67.7%) and cats (11.8%) were the animals most frequently reported and exposure category III was most common (88.6%). Approximately equal numbers of callers were advised active management of administering rabies PEP and conservative management of withholding PEP. This did not seem to be affected by the exposure category related to the call.

**Conclusion:**

This analysis shows the ongoing demand by HCWs for technical support regarding patient management following potential exposure to rabies. Gaps in HCWs rabies knowledge provide unique learning points on guiding training to achieve the goal of eliminating dog-mediated human rabies deaths by 2030.

## Introduction

Rabies is a neglected viral zoonotic disease caused by the *rabies lyssavirus*, of the genus *Lyssavirus*, family *Rhabdoviridae*. Human transmission occurs via the introduction of virus-laden saliva of a rabid animal through breaches in skin or contact with mucous membranes. The rabies virus spreads via peripheral nerves to the central nervous system causing a fatal encephalitis once symptoms develop. Rabies can be prevented through the administration of post-exposure prophylaxis (PEP), which following thorough wound washing, includes the administration of the rabies vaccine with or without the addition of the rabies immunoglobulin (RIG).^1^

Around 59 000 deaths occur annually worldwide due to rabies in humans.^2^ Approximately 96% of these deaths happen in Africa and Asia^2^ where a disproportionately high number occur in rural areas and up to 50% of people exposed are < 16 years.^2,3^ Over 95% of human rabies cases are due to exposure to rabid domestic dogs.^1,2,3,4,5^ Rabies can, however, affect all mammalian species, including many domestic livestock and wildlife species. Small rodents such as rats and mice are not typical rabies reservoirs.^1,4,5^ Other low-risk species include baboons and monkeys.

In South Africa (SA), both domestic and wildlife species , namely the domestic dog, black-backed jackal, bat-eared fox and various mongoose species, serve as reservoirs of the disease.^4,5^ Cases of rabies in domestic dogs have been reported mainly (but not exclusively) in areas in the eastern half of SA including locations in the Eastern Cape, KwaZulu-Natal, Limpopo and Mpumalanga provinces.^6,7^ Rabies in other species is reported from all provinces.^5^ Other lyssaviruses that may also cause clinical rabies have been reported in SA. These include the so-called rabies-related lyssaviruses, namely Lagos bat, Duvenhage and Mokola lyssaviruses.^4,8,9^ These are described primarily from specific species of bats (both insectivores and frugivores) but Mokola virus has to date not yet been reported from bats and the reservoir host of this virus remains unclear. A novel lyssavirus, Matlo lyssavirus has also been described from insectivorous bats in SA.^8^ Human cases of rabies in SA associated with these viruses have been limited to two cases of Duvenhage virus infection.^9^ Despite reports of rabies in numerous other animal species, domestic dogs still pose the greatest rabies risk to the SA population.^7^ In SA, the vaccination of both domestic dogs and cats is required through the Animal Health Act, 2002.^10^ Although domestic cats have not been frequently associated with human rabies cases, vaccination of these animals is recommended given their close contact with humans.^11^ The recommended vaccination schedule to prevent rabies in animals in SA includes vaccination at the ages of three and seven months, again 12 months later and then every three years.^12^

A study by Weyer et al.^7^ regarding the epidemiology of human rabies in SA from 2008 to 2018 revealed that an average of 10 laboratory-confirmed cases are reported annually, with more than 50% younger than nine years of age. The vast majority are linked to exposures to rabid dogs and many were cases in which deviations from PEP protocols were observed.^7^ The SA national guideline^4^ aligns with the *World Health Organization* (*WHO*) *guide for rabies pre- and post-exposure prophylaxis in humans,*^13^ recommending that all individuals exposed to an animal should be assessed and managed based on their exposure risk. This risk assessment should consider the animal species, behaviour and vaccination status, geographical location and rabies endemicity and category of exposure.^4,13^ Although the vaccines used on animals in SA are known to be effective for periods exceeding three years, greater reliance should be placed on the animal’s clinical picture.^4,14^ When exposure to a possibly rabid animal occurs, the exposure category should be assessed to guide management. There are three categories of exposure that have been outlined in Table 1 along with their corresponding recommended management.^4,13^

**TABLE 1:** Categories of exposure and recommended post-exposure prophylaxis.

Category	Description	Management
I	No direct contact with animal In the presence of a rabid animal Touching, petting or feeding Licks on intact skin	No PEP if history is reliable
II	Direct contact but no bleeding Nibbling uncovered skin Minor scratches or abrasions without bleeding	Wound management Full course of rabies vaccine (four divided doses on days 0, 3, 7, 14–28)
III	Direct contact with breach of skin, already broken skin, mucosal membranes or any amount of bleeding; all bat exposures	Wound management Full course of rabies vaccine Rabies immunoglobulin (RIG)

PEP, post-exposure prophylaxis.

The rabies vaccine in humans offers long-lasting immunity, therefore following a suspected exposure, previously vaccinated individuals should receive a two-dose vaccine booster, but no RIG. Repeated PEP is not recommended in the event of an exposure within three months of completion of PEP.^4,13,14^ Rabies immunoglobulin cannot be given to people who delay presentation beyond seven days of receiving the vaccine.^4,13^ Management varies for special groups, of note, people with immunodeficiencies require full PEP irrespective of category or vaccination history. Full PEP is recommended for all bat exposures as bites are often minor and unrecognised. Pre-exposure prophylaxis (PrEP) is recommended for individuals at high or continuous risk of exposure because of their occupation (e.g. veterinarians, laboratory workers), hobbies (e.g. bat enthusiasts) or travel to endemic areas where PEP may be inaccessible.^4,13,14^

Whilst further research into treatment strategies for rabies is underway, the emphasis remains on the provision of timeous and complete PEP, dog vaccinations and improved rabies awareness as the priority interventions to prevent and eliminate the disease.^15^ An Ethiopian study by Beyene et al.^16^ on health-seeking behaviour following animal exposure found that treatment compliance was more likely amongst individuals with higher incomes and easier access to healthcare facilities. However, health education of and by healthcare workers (HCWs) at the point of care regarding the importance of vaccination was emphasised by Tran et al.^17^ in their study in Vietnam, as a potential protective factor in this context.

The National Institute for Communicable Diseases (NICDs) is a public health institute in SA which, amongst other functions, hosts the national rabies reference unit. The NICD provides technical support to all HCWs about infectious diseases through the clinician hotline.^18^ This 24-hour telephonic service, staffed on a rotational basis by various specialists and medically qualified staff, assists with assessment and management advice and may serve as a component of event-based surveillance.^18^ The hotline provides immediate access to expert advice, supplemented by up-to-date evidence based on national and international guidelines.

Whilst no study has been published analysing the data of the NICD clinician hotline, various internal publications have observed rabies-related queries to be common. Common themes include the rabies risk of rodent bites, managing saliva splash injuries and the post-mortem specimen collection of a rabies suspect.^19,20,21^ Comparative research looking at a rabies-specific hotline set up in Chad observed receiving calls mainly from non-HCWs and from rural areas. Most callers sought advice in accordance with category II or III injuries mainly from dogs and cats.^22^

Weyer et al.’s findings of inconsistent application of protocols in confirmed rabies cases in SA guided the aim of this study.^7^ This was to analyse all queries received by the NICD clinician hotline related to rabies PEP from 2016 to 2019 thereby obtaining information of community and HCW knowledge gaps regarding the current protocols and management of suspected rabies exposures.

The specific objectives were (1) to quantify the number of calls received per year, the types and sector of callers and the provinces of callers; (2) to describe the age groups of the people exposed, the animals involved in the exposure, the exposure category and the reported ownership and vaccination-status of the animals involved; and (3) to describe the treatment advice given to callers based on the exposure category.

## Methods

### Study design

A retrospective descriptive review of secondary data was performed.

### Study population

All queries received by the NICD clinician hotline are recorded by the doctor on-call onto an access controlled query database; a secure web-based programme was designed for standardised electronic capturing.

Data from all queries received on the hotline related to rabies PEP in humans following animal exposures from 01 January 2016 to 31 December 2019 were reviewed. Queries that related to diseases other than rabies, related to rabies, but not rabies PEP, follow-up calls and human-bite related calls, were excluded. No recruitment of participants was necessary.

### Data collection and management

On the query database, the variables ‘related disease’, ‘province’, ‘caller type’ and ‘sector’ are recorded in drop-down menus. ‘Scenario description’ and ‘description of action taken’ fields are free-text boxes, narrating the details of the scenario and advice given, respectively. The complete data set was exported onto a Microsoft^®^ Excel spreadsheet.

Three members of the study team were allocated queries from one year each. The remaining records were divided equally amongst this team. Each query was reviewed. Duplicate queries were excluded on review of multiple variables. The variables ‘related disease’, ‘scenario description’ and ‘description of action taken’ were reviewed to exclude queries, which were not part of the study population.

Information regarding exposure history from the ‘scenario description’ narrative was reviewed, extracted and coded as new variables including ‘patient age group’, ‘animal type’, ‘ownership-’ and ‘vaccination-status’ and ‘exposure category’. The ‘description of action taken’ narrative was reviewed and a variable was created and coded to describe the advice that was given to the caller by the doctor on-call. These were grouped into management options that were grouped further into management categories of ‘active’ management in favour of administering PEP and ‘conservative’ management that advised against PEP use. Any uncertainty of coding was discussed between the three study team members.

### Data analysis

Data analysis was performed using Microsoft^®^ Excel. Descriptive statistics (frequencies and percentages), tables and charts were used to present and describe the data.

The number of queries received per year, caller types and sector of callers and the provincial origin of queries were quantified. Exposures were described based on the age groups of people exposed, types of animals involved in the exposure, exposure category and the ownership- and vaccination-status of the animals involved. The treatment advice given to callers was then described in relation to the exposure category that was reported.

### Ethical considerations

Ethical clearance was obtained within a previous ethics approval of ‘essential communicable disease surveillance and outbreak investigation activities of the National Institute for Communicable Diseases’ by the Human Research Ethics Committee (Medical) of the University of the Witwatersrand (reference M160667). Gatekeeper permission to access the NICD database was obtained from the NICD.

## Results

### Characteristics of queries

A total of 4655 queries were received from 01 January 2016 to 31 December 2019. Queries concerning rabies accounted for 62.6% (*n* = 2914). Of these, 2461 (84.5%) were related to rabies PEP and 453 (15.0%) to other rabies-related issues including rabies PrEP, testing for suspected human rabies, follow-up rabies PEP queries and rabies PEP-related queries following human bites. A total of 1741 queries related to diseases other than rabies included calls about malaria, viral haemorrhagic fevers and the 2017–2018 national listeriosis outbreak. The characteristics of the 2461 rabies PEP queries are summarised in Table 2.

**TABLE 2:** Characteristics of rabies post-exposure prophylaxis queries to the National Institute for Communicable Diseases clinician hotline from 01 January 2016 to 31 December 2019.

Characteristic	*n*	%
**Number of queries by year**	2461	
2016	501	20.4
2017	655	26.6
2018	701	28.5
2019	604	24.5
**Caller type**	2437	
HCW	2313	94.9
*Public sector*	448	19.4
Doctor	269	60.0
Nurse	123	27.5
Provincial surveillance	13	2.9
Laboratorian	1	0.2
Other HCW	42	9.4
*Private sector*	1865	80.6
Doctor	1558	83.5
Nurse	252	13.5
Provincial surveillance	1	0.1
Laboratorian	3	0.2
Other HCW	41	2.2
Non-HCW	124	5.1
**Province**	2443	
Gauteng	912	37.3
KwaZulu-Natal	875	35.8
Western Cape	201	8.2
Free State	144	5.9
Eastern Cape	112	4.6
Mpumalanga	70	2.9
North West	64	2.6
Limpopo	29	1.2
Northern Cape	21	0.9
External to SA	15	0.6
**Age group of exposed patients**		
Child	697	28.3
Adult	1431	58.1
Not stated	321	13.0
Multiple people	12	0.5

SA, South Africa; HCW, healthcare workers.

Call numbers progressively increased from 2016 (*n* = 501) to 2018 (*n* = 701); however, 2019 recorded the lowest number of calls (*n* = 604) (Table 2). The proportion of rabies-PEP-related calls remained consistent over the years in the study period. Where caller type and sector were known (*n* = 2437), majority of callers were HCWs (94.9%), of which 80.6% were from the private health sector. Other HCW-callers included the surveillance department within provincial or district departments of health and laboratory staff. Non-HCWs accounted for 5.1% of callers and included members of the public and journalists.

The provincial origin of calls was known in 2443 calls. Queries were received from all nine provinces; however, over 70.0% of calls originated from Gauteng (37.3%) and KwaZulu-Natal (35.8%). Calls from outside SA made up 0.6% of all calls.

### Characteristics of exposure

Two-thirds of queries about patients with documented ages were adults (Table 2). Thirteen per cent of calls did not document an age and 0.5% of calls referred to multiple people exposed to the same animal.

**FIGURE 1:**
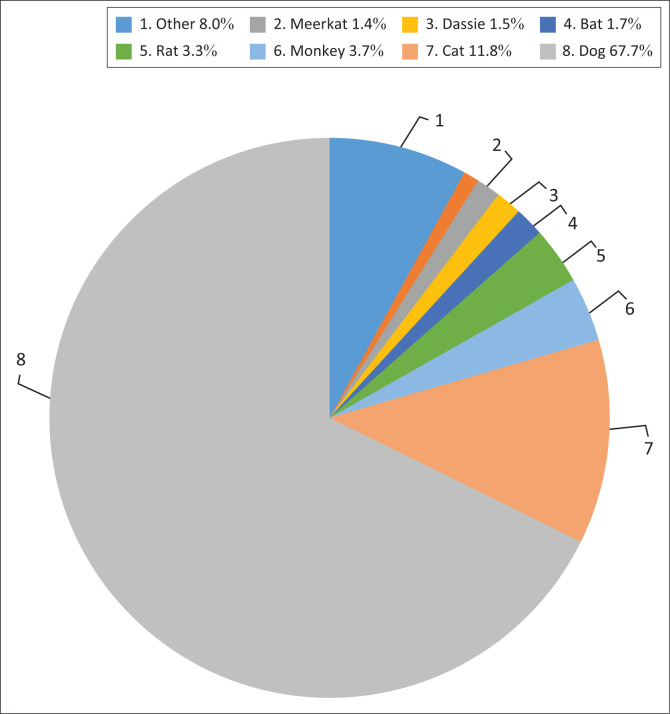
Proportion of queries per animal exposure.

From the rabies PEP-related queries, 50 different animals were reported to be implicated in exposures with humans. The vast majority of queries observed exposure to dogs (67.7%) and cats (11.8%). Other commonly encountered animals, representing those reported in more than 1.0% of calls each, included monkeys, rats, bats, rock hyrax (dassies) and meerkats. Less commonly encountered animals in the ‘other’ category included cows, rabbits, mice, mongooses, pigs and horses (Figure 1).

**FIGURE 2:**
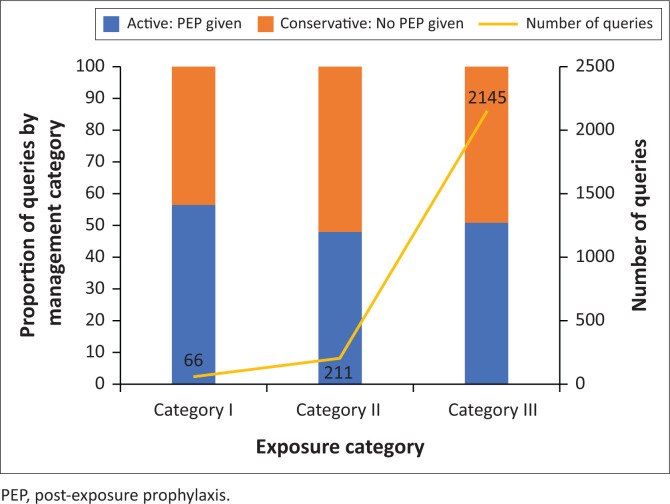
Number of queries and management advise per exposure category, 01 January 2016 to 31 December 2019.

Of the 2461 queries, the category of exposure was described in 2422 queries. Most queries were related to category III exposure scenarios (*n* = 2145, 88.6%) (Figure 2).

Table 3 summarises the vaccination- and ownership-status of animals involved in human exposure. Most exposures were related to owned animals. The vaccination status was known in 43.6% (*n* = 1072) of queries, of which just over half (*n* = 558) reported vaccination against rabies. The vaccination status was observed to be unknown to the caller in 37.5% (*n* = 923) of queries and not recorded in the query database in 18.9% (*n* = 466) of queries.

**TABLE 3:** Distribution of vaccination status amongst animals of varying ownership status, 01 January 2016 to 31 December 2019.

Animal ownership	Animal previously vaccinated				Total
	No	Yes	Unknown	Not stated	
Owned	241	556	329	339	1465
Wild	258	1	50	34	343
Stray	0	0	447	0	447
Unknown	0	0	65	0	65
Not stated	15	1	32	93	141

**Total**	**514**	**558**	**923**	**466**	**2461**

**FIGURE 3:**
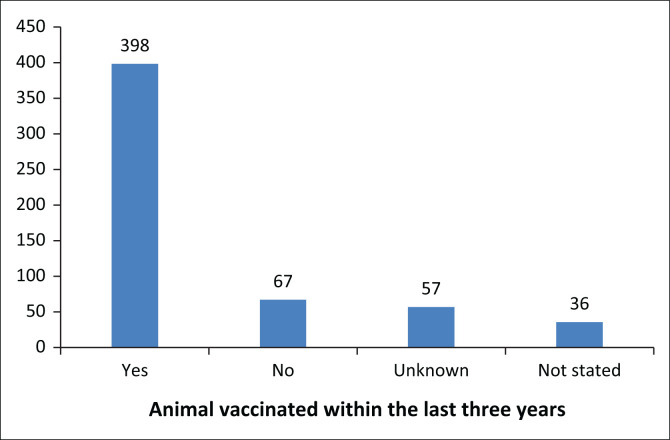
Completeness of vaccination for animals known to be vaccinated.

On further analysis of the 558 queries involving animals with known positive vaccination histories (Figure 3), 71.3% (*n* = 398) were reported to be up to date (vaccination within the last three years), with 12.0% not up to date. The last vaccination date of the remaining 16.7% were either unknown to the caller or not recorded in the query database.

### Types of treatment advised

The categories of management advice provided per exposure category are illustrated as a proportion of queries in Figure 2. For each exposure category, approximately equal proportions of queries were responded to with active and conservative management advice.

## Discussion

### Characteristics of calls

Most queries received during the study period were rabies related, majority of which were PEP-specific. This highlights the continued need for the hotline as an aid in patient management.

The lower number of calls received in 2019 may be because of improved knowledge of rabies management through campaigns and training or improved uptake and understanding of rabies management guidelines. However, this may also be because of reduced exposures of patients to animals, reduced presentation following these exposures or a decline in knowledge of the hotline to assist clinical management.

Doctors and nurses were the most frequent callers to the hotline. Just over 80% of HCWs were from the private sector. This private-public sector discrepancy is thought to be because of protocol differences between sectors but may also be as a result of independent practitioners requiring support that is otherwise unavailable in the private sector.

**FIGURE 4:**
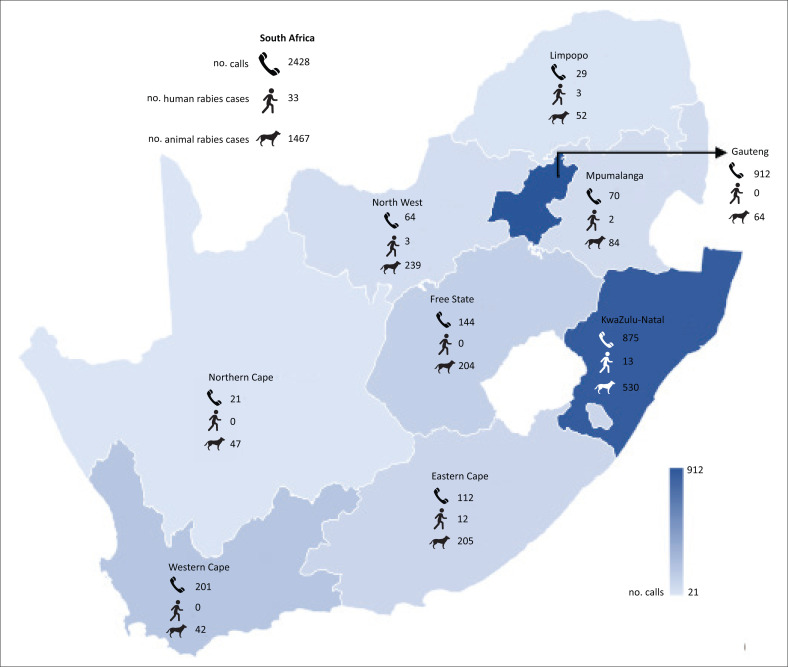
Provincial distribution of calls received, confirmed human^26^ and animal^6^ rabies cases from 01 January 2016 to 31 December 2019.

High numbers of rabid animals in a province may lead to increased animal exposures and in turn, more hotline queries. However, should exposed persons not access medical attention, low call numbers would be expected. Figure 4 shows the provincial distribution of queries and the number of human and animal cases over the study period. The numbers from each province were varied and no obvious patterns could be seen between the number of queries and the number of human and animal cases per province over the same period. Low query numbers from some provinces may indicate a low presentation of animal exposures to healthcare facilities, lack of awareness of the NICD clinician hotline or increased comfort of HCWs with rabies management in these provinces.

### Characteristics of exposures

Inconsistencies in the documentation of age made the accurate analysis of the data by age group difficult (numerical age vs. the term ‘adult/child’). There were 558 queries involving children between 0 and 14 years old and an additional 139 labelled with the term ‘child’. Children therefore accounted for 32.8% of patients. In Africa and Asia, children less than 16 years account for half of all rabies exposures^3^ and the SA findings note that over half of human rabies cases occur in children less than nine years.^7^ This discrepancy of reported exposures mainly involving adults could indicate poor health-seeking behaviour on behalf of children for rabies PEP or children not reporting animal exposures to caregivers, both of which increase the risk of rabies in children.

Most queries involved exposure to domestic dogs and cats, which aligns with established evidence that these species pose the greatest threat of rabies transmission to humans.^1,7^ Furthermore, it emphasises the need to educate communities about safe animal handling and animal vaccinations to minimise risk.^15^

The high-risk exposure to bats accounted for 1.7% of the queries, despite management being highlighted in the national rabies guideline.^4^ Queries were also received about exposure to non-mammals such as birds and reptiles that carry no risk of rabies and low-risk rodent species.^1,4,5^ The frequency of these calls may promote a revision of the guidelines to outline the risk of rabies by species.

Almost 40% of owned animals were unvaccinated or had an unknown vaccination status. Whilst this is not wholly representative of vaccination status of owned animals in the country, it highlights the need for a continued drive to promote animal vaccination as per the *Animal Health Act*.^10^

### Types of treatment advised

Active and conservative management categories have been advised with similar proportions across exposure categories. Category I queries that were advised active management were related to confirmed or highly suspected rabies in the animal of exposure, the animal having had contact with another confirmed rabid animal, or the patient being in a high-risk occupation. Almost half of all the callers querying about managing category II and III exposures were advised to manage the patient conservatively. This included queries where the animal was owned by the patient and was not behaving strangely, had an up-to-date vaccination status, could be observed for signs of rabies or was not a species likely to transmit rabies.

Rabies PEP is required if a risk assessment indicates the possibility of rabies exposure. As seen here where active and conservative management were advised with similar proportions across exposure categories, the risk assessment should not be based on the exposure category alone but should include the behaviour and health status of the animal, animal species, geographical area where the exposure took place and the animal vaccination status. The rabies vaccine, and particularly RIG, are in limited supply in the country; therefore, an appropriate risk assessment is needed to ensure their proper use to reduce wastage. The NICD plays an important gate keeping role for rabies. In 2021, the NICD confirmed 19 human rabies cases.^24^ The hotline should be contacted for expert advice in the prevention of rabies in humans, especially in complex exposures.

Other factors in clinical scenarios were recognised to complicate management; these included queries on how to manage previously vaccinated patients, how to interpret rabies antibody titres, how to modify the PEP schedule for those intermittently lost to follow-up and how to adjust management based on new information regarding the animal. This illustrates the multiple factors to be considered in risk assessment and management. These factors transcend an inflexible algorithm of management based exclusively on exposure category and this may form part of why HCWs require assistance with formulating management plans.

### Strengths and limitations

The central position of the NICD as an institute providing technical support makes the query database of the NICD clinician hotline a rich source of information, which spans call data from numerous settings across the country. Whilst this data is not nationally representative, some knowledge and exposure trends may still be extracted.

The main challenges faced whilst working with the query database were of varied data quality because of inconsistent types and formats of data recorded and incomplete recording of important information. Data cleaning, de-duplication and coding of data was complex and time-consuming.

### Recommendations

As the query database is not designed specifically for rabies, important rabies-related data were often missing. A more directed rabies module within the data system could include separate fields for the patient’s age and vaccination history, animal species and vaccination status, exposure category and specific management options. This would allow for clearer and more quantifiable database analysis in the future whilst contributing to rabies surveillance.

Improvements to current knowledge regarding managing potential rabies exposures may be influenced by improvements to rabies guidelines. This should focus on clearly defining the assessment of rabies risk and the management thereafter based on all factors affecting these rather than the exposure category-driven algorithm that currently exists. Rabies guidelines should be presented in easily understandable formats and should be shared across various physical and digital platforms^25^ and be disseminated through training opportunities that incentivise HCWs by providing continual professional development points.

Whilst this study is not representative of all potential rabies exposures in SA, some trends seen may be suggestive of gaps in the rabies control programme which should be addressed. This includes a suboptimal number of vaccinated dogs and cats and more so, gaps in community knowledge on early health-seeking for assessment following animal exposure, as well as follow-up adherence.

Suggested future studies would include the follow up and review of outcomes in patients who sought rabies management advice on the NICD hotline.

## Conclusion

The more than 2400 rabies queries over four years, to the NICD clinician hotline, illustrate the demand by HCWs for technical support regarding patient management. Trends about the origin of queries, animal vaccination status and HCW rabies knowledge provide unique learning points in aiding prevention strategies and improving management guidelines all with the aim of eliminating human rabies.

## References

[1] World Health Organization. WHO expert consultation on rabies, third report [document on the Internet]. 3rd ed. World Health Organization – Technical Report Series No. 1012. Geneva: WHO Press; 2018 [cited 2020 Nov 11]. Available from: https://apps.who.int/iris/bitstream/handle/10665/272364/9789241210218-eng.pdf?sequence=1&isAllowed=y

[2] Hampson K, Coudeville L, Lembo T, et al. Estimating the global burden of endemic canine rabies. PLoS Negl Trop Dis. 2015;9(4):1–20. https://doi.org/10.1371%2Fjournal.pntd.0003709 10.1371/journal.pntd.0003709 PMC4400070 25881058

[3] Knobel DL, Cleaveland S, Coleman PG, et al. Re-evaluating the burden of rabies in Africa and Asia. Bull World Health Organ. 2005;83(5):360–368. 15976877 PMC2626230

[4] Bishop GC, Durrheim DN, Kloeck PE, et al. Rabies: Guide for the medical, veterinary and allied professions [homepage on the Internet]. 2nd ed. In: Blumberg LH, Weyer J, Pienaar H, Markotter W, 2008 RAG, editors. Pretoria: Government Printer; 2010 [cited 2021 Mar 10]. Available from: https://www.nda.agric.za/docs/GenPub/rabiesB5.pdf

[5] Weyer J, Blumberg LH. Rabies: Prevention and management. In: MIMS handbook of general medicine – Volume 1 [homepage on the Internet]. Johannesburg: Tiso Blackstar Group; 2019 [cited 2020 Nov 17]. Available from: https://anyflip.com/enio/wdtk/basic/151-200

[6] Department of Agriculture Land Reform and Rural Development (DALRRD). Disease database: Confirmed animal rabies cases in SA, 2016–2019 [homepage on the Internet]. [cited 2021 Jan 10]. Available from: http://webapps.daff.gov.za/VetWeb/dieaseDatabase.do;jsessionid=213a10c7e83e3088773b6750d47b

[7] Weyer J, Dermaux-Msimang V, Grobbelaar A, et al. Epidemiology of human rabies in South Africa, 2008 – 2018. S Afr Med J. 2020;110(9):877–881. https://doi.org/10.7196%2Fsamj.2020.v110i9.14324 32880272 10.7196/SAMJ.2020.v110i9.14324

[8] Coertse J, Grobler CS, Sabeta CT, et al. Lyssaviruses in insectivorous bats, South Africa, 2003–2018. Emerg Infect Dis. 2020;26(12):3056–3060. https://doi.org/10.3201%2Feid2612.203592 33219800 10.3201/eid2612.203592 PMC7706942

[9] Paweska JT, Blumberg LH, Liebenberg C, et al. Fatal human infection with rabies-related Duvenhage virus, South Africa. Emerg Infect Dis. 2006;12(12):1965–1967. https://doi.org/10.3201%2Feid1212.060764 17326954 10.3201/eid1212.060764 PMC3291369

[10] South Africa. Animal Health Act No. 7 of 2002 [statuette on the Internet]. [cited 2020 Dec 20]. Available from: https://www.gov.za/sites/default/files/gcis_document/201409/a7-02.pdf

[11] Grobbelaar AA, Blumberg LH, Dermaux-Msimang V, et al. Human rabies associated with domestic cat exposures in South Africa, 1983–2018. J S Afr Vet Assoc. 2020;91(0):1–4. https://doi.org/10.4102%2Fjsava.v91i0.2036 10.4102/jsava.v91i0.2036 PMC7433215 32633988

[12] Government of South Africa. The truth about rabies [document on the Internet]. 2012 [cited 2021 Apr 8]. Available from: https://www.gov.za/truth-about-rabies#:~:text=

[13] World Health Organization. WHO guide for rabies pre- and post-exposure prophylaxis in humans [document on the Internet]. 2014 [cited 2020 Nov 20]. Available from: https://www.who.int/rabies/PEP_Prophylaxis_guideline_15_12_2014.pdf

[14] Blumberg LH, Weyer J, Frean J, Ogunbanjo GA. Rabies: An evidence-based approach to management. S Afr Fam Pract. 2007;49(7):35–40. https://doi.org/10.1080%2F20786204.2007.10873599

[15] World Health Organization, Food and Agriculture Organization of the United Nations, World Health Organisation for Animal Health, Global Alliance for Rabies Control. Zero By 30: The global strategic plan to end human deaths from dog-mediated rabies by 2030 [document on the Internet]. Geneva; 2019 [cited 2021 Mar 02]. Available from: https://apps.who.int/iris/bitstream/handle/10665/328053/WHO-CDS-NTD-NZD-2019.04-eng.pdf?sequence=1&isAllowed=y

[16] Beyene TJ, Mourits MCM, Revie CW, Hogeveen H. Determinants of health seeking behaviour following rabies exposure in Ethiopia. Zoonoses Public Health. 2018;65(0):443–453. https://doi.org/10.1111%2Fzph.12458 29524317 10.1111/zph.12458

[17] Tran CH, Afriyie DO, Pham TN, et al. Rabies post-exposure prophylaxis initiation and adherence among patients in Vietnam, 2014–2016. Vaccine. 2019;37:A54–A63. https://doi.org/10.1016%2Fj.vaccine.2019.01.030 30723063 10.1016/j.vaccine.2019.01.030

[18] National Institute for Communicable Diseases. Division of public health surveillance and response: Objectives[homepage on the Internet]. [cited 2021 Apr 8]. Available from: https://www.nicd.ac.za/centres/division-of-public-health-surveillance-and-response/

[19] National Institute for Communicable Diseases. Frequently asked questions to the NICD 24-hour hotline. Communicable Dis Communiqué [serial online]. 2018 [cited 2021 Mar 03];17(12):7. Available from: https://www.nicd.ac.za/wp-content/uploads/2019/03/NICD-Communicable-Diseases-Communique_Dec-2018_final.pdf

[20] National Institute for Communicable Diseases. Frequently asked questions on the NICD 24-hour hotline. Communicable Dis Communiqué [serial online]. 2018 [cited 2021 Mar 05];17(9):9. Available from: https://www.nicd.ac.za/wp-content/uploads/2018/09/NICD-Communicable-Diseases-Communique_Sept2018_final.pdf

[21] National Institute for Communicable Diseases. Frequently asked questions to the NICD 24-hour hotline. Communicable Dis Communiqué [serial online]. 2019 [cited 2021 Feb 02];18(1):8. Available from: https://www.nicd.ac.za/wp-content/uploads/2019/03/NICD-Communicable-Diseases-Communique_Jan2019_final.pdf

[22] Mbaipago N, Mindekem R, Madjiadinan A, et al. Short communication on the use of a free rabies hotline service in Chad. Acta Trop. 2020;206:105446. 10.1016/j.actatropica.2020.105446 32184101

[23] World Health Organization. Outbreak communication planning guide [homepage on the Internet]. Geneva: WHO Document Production Services; 2008 [cited 2021 Jan 10]. Available from: http://www.who.int/ihr/elibrary/WHOOutbreakCommsPlanngGuide.pdf

[24] National Institute for Communicable Diseases. An update on rabies in South Africa. Communicable Dis Communiqué [serial online]. 2022 [cited 2020 Dec 01];21(1):3. Available from: https://www.nicd.ac.za/wp-content/uploads/2022/01/NICD-Monthly-Communique-January.pdf

[25] World Health Organization. WHO guideline: Recommendations on digital interventions for health system strengthening [homepage on the Internet]. 2019 [cited 2021 Mar 05]. Available from: https://apps.who.int/iris/bitstream/handle/10665/311941/9789241550505-eng.pdf?ua=1 31162915

[26] National Institute for Communicable Diseases. An update on rabies in South Africa. Communicable Dis Communiqué [serial online]. 2021 [cited 2022 Feb 17];20(1):2. Available from: https://www.nicd.ac.za/wp-content/uploads/2021/01/An-update-on-rabies-in-South-Africa.pdf

